# The Effect of the COVID-19 Pandemic Lockdown on Self-Harm: A Meta-Analysis

**DOI:** 10.31083/AP39868

**Published:** 2025-04-22

**Authors:** Jue Wang, Xueqian Zhang, Hu Deng, Yunlong Tan

**Affiliations:** ^1^Peking University Huilongguan Clinical Medical School, Beijing Huilongguan Hospital, 100096 Beijing, China

**Keywords:** self-injurious behavior, COVID-19, social isolation, pandemics, meta-analysis

## Abstract

**Objective:**

The Coronavirus disease 2019 (COVID-19) pandemic caused a range of mental health problems, particularly self-harm. Lockdowns are the usual methods of responding to these public health emergencies. However, the effect of the COVID-19 lockdown on self-harm remains poorly characterized. This study aimed to investigate the influence of the COVID-19 pandemic on the incidence of self-harm. The findings may inform future policy development and strategies for managing pandemic-related mental health challenges.

**Methods:**

A meta-analysis was conducted using several database searches: APA PsycINFO, Embase, PubMed, Web of Science, CNKI, and Wan Fang. Published studies with data on the incidence of self-harm during visits to medical institutions, before and during the COVID-19 pandemic, were included. The pooled risk ratio (RR) value of self-harm incidence variation before and during the COVID-19 lockdown period, expressed as the comparison of clinical institution visits before and during the pandemic, was calculated.

**Results:**

Fifteen retrospective cohort studies with observational designs involving 253,600 participants were included. The pooled RR value of self-harm incidence variation was 1.386 (95% confidence interval (CI), 1.205–1.595, I^2^ = 58.9%, *p* = 0.002). The subgroup analysis showed that “emergency department type” (*p* = 0.004) and “mean age of the sample” were the sources of the RR values’ heterogeneity (*p* = 0.026).

**Conclusions:**

Our findings suggest that the lockdown during the COVID-19 pandemic was a risk factor for self-harm. Therefore, special attention should be paid to individuals visiting the emergency department and the middle-aged and elderly populations.

**The PROSPERO Registration:**

This study was registered in PROSPERO (CRD42023373026), https://www.crd.york.ac.uk/PROSPERO/view/CRD42023373026.

## Main Points

- The COVID-19 pandemic and associated lockdowns have been linked to an increase in self-harm incidents.
- The study conducted a meta-analysis of 15 retrospective cohort studies, which included 253,600 participants, showing a pooled risk ratio of 1.386 for increased self-harm incidence during the pandemic compared to pre-pandemic levels.
- The analysis revealed that differences in self-harm incidence were influenced by the emergency department type and the mean age of the sample, indicating that these factors contribute to the heterogeneity in risk ratios.
- The findings suggest that lockdown measures may be a risk factor for self-harm, underscoring the need for targeted mental health support.

## 1. Introduction

The Coronavirus disease (COVID-19) pandemic has led to worldwide lifestyle changes. During the pandemic, lockdown measures were implemented worldwide to stop the spread of COVID-19. These lockdowns caused a range of mental health problems [1, 2, 3]. The combination of physical health risks, social isolation, economic challenges, and disruptions to daily life caused by the pandemic has led to an increase in various mental health conditions, such as self-harming behavior. The World Health Organization (WHO) defines this as an intentional action that results in self-harm, either through non-habitual behavior or excessive substance ingestion, to achieve desired physical changes [4].

A meta-analysis of data from 40 countries found that the overall lifetime prevalence of self-harm was 16.9%. The average age at self-harm initiation was approximately 13 years, with cutting being the most common type (45%). Suicidal ideation (risk ratio (RR): 4.97) and suicide attempts (risk ratio: 9.14) were significantly higher among young people who engaged in self-harming behaviors than in elderly people. Previous studies have shown that suicide and self-harm rates may increase during and in the aftermath of a pandemic [5, 6].

Lockdown policies during the COVID-19 pandemic imposed restrictions on the established patterns of social and economic life. Evidence from other studies suggests that lockdown measures would reduce the effective reproduction rate of the virus in several countries [7]. However, the lockdown policy might have aggravated the incidence of self-harm, which may have negative political consequences that public health authorities should consider, including the prolonged or disproportionate imposition of restrictions on personal freedoms and civil liberties and the suspension of democratic procedures and safeguards. Therefore, we should elaborate on variations in self-harm incidence before and during the COVID-19 lockdown.

Isolation may mask mental health issues, which may lead to a significant increase in self-harm [8, 9]. Health can also be affected by conflict as a result of authorities coercing and sanctioning households and communities that are unable or unwilling to comply with lockdown measures [7]. The COVID-19 lockdown has affected the operation of the health system by increasing physical and financial constraints on access to healthcare, diverting attention and resources to COVID-19, and leading patients to stay away from hospitals for fear of contracting COVID-19 [7].

Previous studies have shown an increase in the incidence of self-harm in hospital [10, 11, 12, 13] emergency departments and nonhospital emergency departments [14] during the COVID-19 pandemic. Interestingly, the lockdown measures might mediate the reduction in psychiatric emergency presentations [15]. Moreover, self-harm incidence was correlated with age. Previous studies showed that the number of adolescents deliberately harming themselves during the COVID-19 pandemic has risen, which has garnered attention [16], particularly regarding adolescent girls [17, 18, 19]. Therefore, it is worth exploring the type of clinical setting and age underlying self-harming behavior.

Some studies have demonstrated that the COVID-19 pandemic and the related lockdown may serve as risk factors contributing to the increasing incidence of self-harm, and age and the selection of clinical institutions might enhance this relationship. Self-harm exacerbates the emotional or physical pain endured by an individual over time and has a profound negative impact on an individual’s social well-being across all aspects of life. However, the utilization of the risk ratio as an effective measure for comparing self-harm incidence before and during the COVID-19 lockdown period has been overlooked. Therefore, our study aimed to conduct a comprehensive meta-analysis of existing literature to examine this issue.

## 2. Methods

### 2.1 Search Strategy and Selection Criteria 

We searched APA PsycINFO, Embase, PubMed, Web of Science, CNKI, Wan Fang, and VIP for English-language sources published between Jan 1, 2020, and April 30, 2022. We searched the literature with the following keywords: (“COVID-19” or “Corona Virus Disease 2019”) AND (“NSSI” or “None suicide self-injury” or “Self-harm” or “Self-injury” or “Self-injurious behavior” or “Deliberate self-harm” or “DSH” or “Self-cut”). References to related research have also been reviewed in the correlative studies.

Two researchers independently evaluated the abstracts for related research that satisfied the above search strategy, and the full-text articles were further assessed by two researchers to determine whether they met the inclusion criteria.

The inclusion criteria were as follows: (1) Data from related articles published in English by clinical institutions; (2) All articles disclosed numbers of self-harm and other visits before and during the COVID-19 lockdown period; (3) Articles must have specified self-harm definitions according to WHO or ICD-10 criteria.

The exclusion criteria were as follows: (1) The total number of visits and number of self-harm visits were not disclosed; (2) Duplicate records; (3) Not available in full text; (4) Empirical research that included conference abstracts, case reports, reviews, expert comments, letters, and dissertations.

This review followed the Preferred Reporting Items for Systematic Reviews and Meta-Analyses guidelines (PRISMA). This study was registered with PROSPERO (CRD42023373026).

### 2.2 Quality Assessment for Included Studies

We used the National Institute of Health Quality Assessment Tool for Observational Cohort and Cross-Sectional Studies to assess study quality (**Supplementary Table 1**). Each item was scored as 0 (unmet criterion) or 1 (met criterion) based on study quality. There were ten items in total, and the final scores ranged from 0 to 10 (Table 1, Ref. [10, 11, 12, 15, 20, 21, 22, 23, 24, 25, 26, 27, 28, 29, 30] and **Supplementary Table 2**). We considered a score of more than 6 as qualifying.

**Table 1.** **Characteristics of studies included in the meta-analysis**.

Author (Year)	Region	Country	Before COVID-19 (total)	During COVID-19 (total)	Before COVID-19 (self-harm)	During COVID-19 (self-harm)	Age	EDT	SES	Statistic	Definition
Hartnett Y, *et al*. (2023) [20]	Europe	Ireland	115,981	51,757	801	437	18–45	Mental Emergency	developed	Prism	ICD-10
John SM, *et al*. (2021) [21]	Asia	India	17,234	14,687	203	179	18–45	Non-Hospital Emergency Department.	developing	SPSS	non-ICD
MacDonald DRW, *et al*. (2020) [22]	Europe	UK	1791	1315	20	22	>45	Non-Hospital Emergency Department.	developed	SPSS	non-ICD
McIntyre A, *et al*. (2021) [23]	Europe	Ireland	760	576	130	119	18–45	Mental Emergency	developed	SPSS	non-ICD
Berger G, *et al*. (2022) [24]	Europe	Switzerland	109	250	33	109	<18	Mental Emergency	developed	SPSS	ICD-10
Trier F, *et al*. (2022) [12]	Europe	Denmark	1159	684	14	16	>45	Hospital Emergency Department.	developed	Stata	non-ICD
Joyce LR, *et al*. (2021) [25]	Others	New Zealand	564	371	35	36	18–45	Mental Emergency	developed	SPSS	non-ICD
Olding J, *et al*. (2021) [26]	Europe	UK	46	30	5	8	18–45	Mental Emergency	developed	Unknown	non-ICD
Shrestha R, *et al*. (2021) [10]	Asia	Nepal	3926	2085	38	55	18–45	Hospital Emergency Department.	developing	SPSS	non-ICD
Bhattaram S, *et al*. (2022) [11]	Asia	India	8654	12,238	64	102	18–45	Non-Hospital Emergency Department.	developing	SPSS	non-ICD
Wong BHC, *et al*. (2022) [15]	Europe	10 Countries	1239	834	612	470	<18	Mental Emergency	developed	Stata	non-ICD
Stevens J, *et al*. (2021) [27]	Europe	UK	981	744	26	43	>45	Hospital Emergency Department.	developed	Prism	non-ICD
Waseem S, *et al*. (2022) [28]	Europe	UK	411	371	11	12	>45	Hospital Emergency Department.	developed	SPSS	non-ICD
Shields C, *et al*. (2021) [29]	Europe	UK	9038	5676	130	118	18–45	Hospital Emergency Department.	developed	Unknown	non-ICD
Díaz de Neira M, *et al*. (2021) [30]	Europe	Spain	64	25	16	9	<18	Hospital Emergency Department.	developed	SPSS	ICD-10

COVID-19, Coronavirus disease 2019; EDT, emergency department type; SES, Social economic status; ICD, International Classification of Diseases.

### 2.3 Data Extraction and Preparation

The following data were extracted from each included study: authors, publication years, study characteristics (e.g., country, area, economic conditions, clinical institution type, statistical methods, and self-harm definition), sample characteristics (e.g., sample size, female ratio, average/mean age, mood disorder incidence), total number of all presentations (number of patients arriving), and number of self-harm presentations before and during the COVID-19 lockdown period. “Female ratio” was calculated as the percentage of females compared to the total number of participants. “Socioeconomic status (SES)” included high-income and low-income countries, categorized based on levels of economic and social development. “Emergency department type (‘EDT’ for short)” was divided into three groups, including Mental Emergency Department, Hospital Emergency Department, and non-hospital emergency department by types of medical visits in the sample population. “Mean age of the sample (Age_n represented for)” was divided into three age groups: “18–45”, “<18”, and “>45”. “Statistic” represented the statistical methods used in the studies. “Definition” meant how the studies defined self-harm, which included both International Classification of Diseases, 10th Revision (ICD-10) and non-ICD-10 measures. Data were independently extracted by two researchers, discrepancies were discussed, and a consensus was reached (Table 1).

RR, also known as the relative risk ratio (RR), was a statistical measure used to quantify the relationship between two groups in a study regarding the likelihood of an event occurring. In this review, RRs were calculated by dividing the self-harm rate during the COVID-19 pandemic by the pre-COVID-19 self-harm rate using a 95% confidence interval (CI). RR = 1.0 indicated no association; RR <1.0 indicated that such a factor might be a protective factor; and RR >1.0 indicated it might be a risk factor.

### 2.4 Statistical Analysis

A random-effects meta-analysis model was performed in R Studio (version 4.2.2), RStudio, Inc., Boston, MA, USA. with the packages “tidyverse”, “meta”, and “metafor” [31]. The random-effects model can provide a more conservative estimate of the overall treatment effect by considering both within-study and between-study variability while also allowing for heterogeneity in effect sizes across studies. In the elementary meta-analysis, the dependent variable was the risk ratio (RR) for self-harm incidence. We conducted sample estimates in R Studio (version 4.2.2) with the package “pwr” [32]. The sample size of the observation presentations was pooled using power calculations (**Supplementary Table 3**). Data were summarized as RRs, and I^2^ and forest plots were used to identify the between-study heterogeneity of RRs under COVID-19 exposure across the included studies.

Sensitivity analysis was performed through an influential analysis by excluding each study to identify potential sources of bias or heterogeneity in the meta-analysis results [33], and the authors agreed on whether to exclude specific studies based on the high variation of the I^2^ value with careful consideration of the studies’ characteristics. After adjusting for inclusion in the final studies, the ultimate forest plot of the RRs was pooled. Based on the considerable heterogeneity in the outcomes, we conducted heterogeneity and sensitivity analyses of the overall group, as well as publication bias analysis. The Q and I^2^ statistics were used to test for heterogeneity. A significant Q statistic (*p* < 0.05) indicated significant heterogeneity. I^2^ values of 0–25%, 25–75%, and >75% represented low heterogeneity, modest inconsistency, and high inconsistency, respectively. If I^2^ was >50%, a random-effects model was used to assess the proportion and accompanying 95% confidence intervals (CIs) [34]. Funnel plots were used to visually show whether publication bias remained, and Egger’s and linear regression tests were applied to quantitatively evaluate publication bias (**Supplementary Figs. 1–6**). If publication bias was observed, the trim-and-fill method was applied to adjust for funnel plot asymmetry and evaluate the influence of bias. Subsequently, subgroup and meta-regression analyses were conducted to explore COVID-19 as an influencing factor in the incidence of self-harm. The subgroups included area, socioeconomic status, emergency department type, age of the target population, statistical method, and self-harm definition. Meta-regressions concerning “sample size”, “self-harm presentations”, and “female ratio” were also conducted.

## 3. Results

### 3.1 Study Selection and Overview of Included Studies

**Fig. 1.**
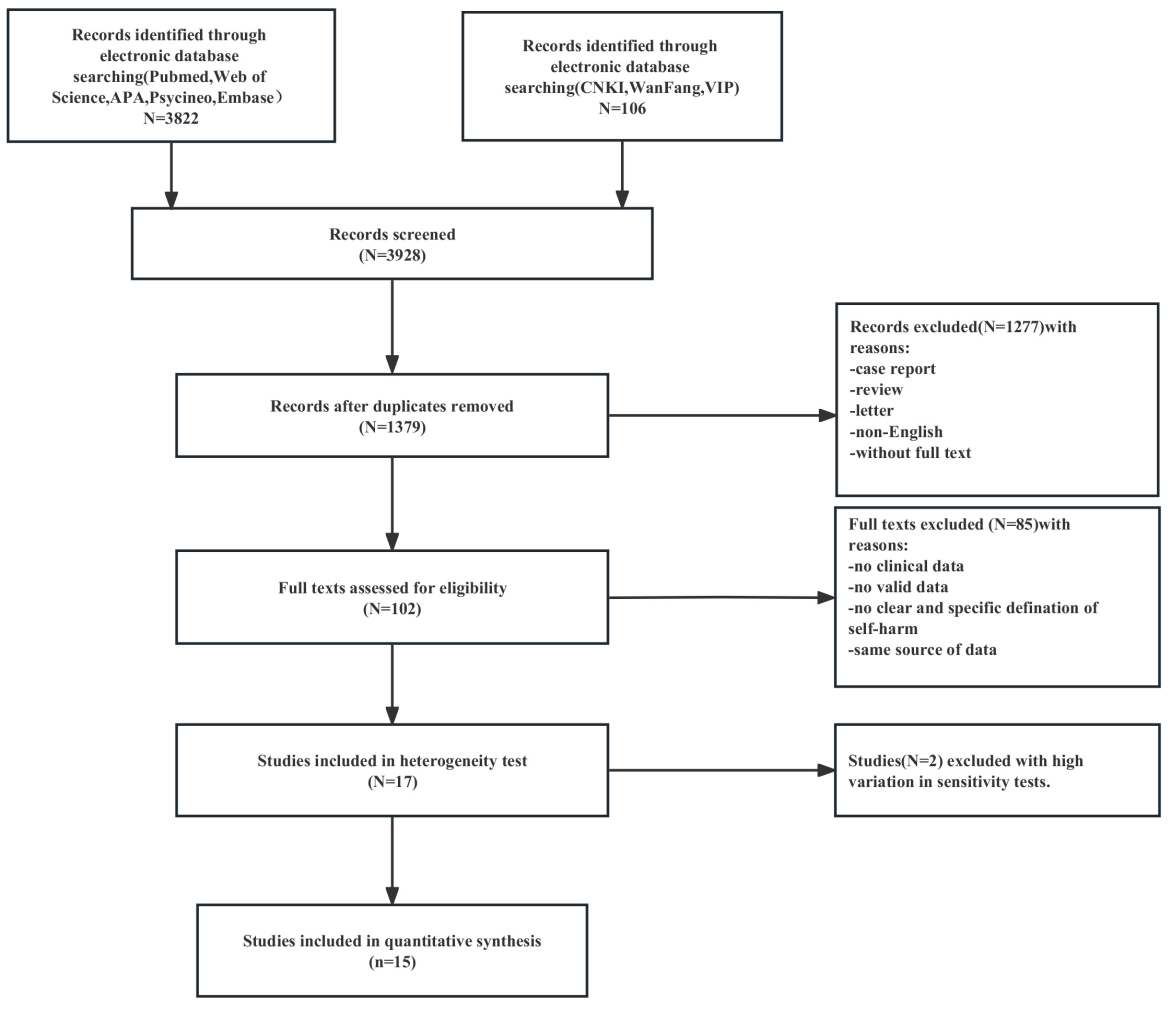
**Meta-analytic study decision tree**.

The initial database search produced 3928 records, with 1379 remaining after duplicates were removed. Screening by title, abstract, and subtitles led to 102 studies, excluding 1277 case reports, reviews, letters, non-English articles, and studies without full text available. After excluding 85 studies with no clinical data, no valid data, duplicate sources of data, and no clear or specific definition of self-harm, 17 studies were included in the heterogeneity test. Following that, 15 studies were included in the meta-analysis, with a total number of self-harm episodes before and during the COVID-19 pandemic of 1330 and 1735, respectively. The total number of medical visits before and during the COVID-19 pandemic was 161,957 and 91,643, respectively (Fig. 1). These 15 studies were observational retrospective cohort studies. Five studies were conducted in the UK; two in Ireland; two in India; one each in Switzerland, Denmark, New Zealand, Nepal, and Spain; and one was a simultaneous study of 10 European countries. The number of self-harm presentations included in the studies ranged from 13 to 1238. In addition, all the studies were based on medical care information.

### 3.2 Meta-Analysis of Self-Harm Incidence before and during the COVID-19 Lockdown

**Fig. 2.**
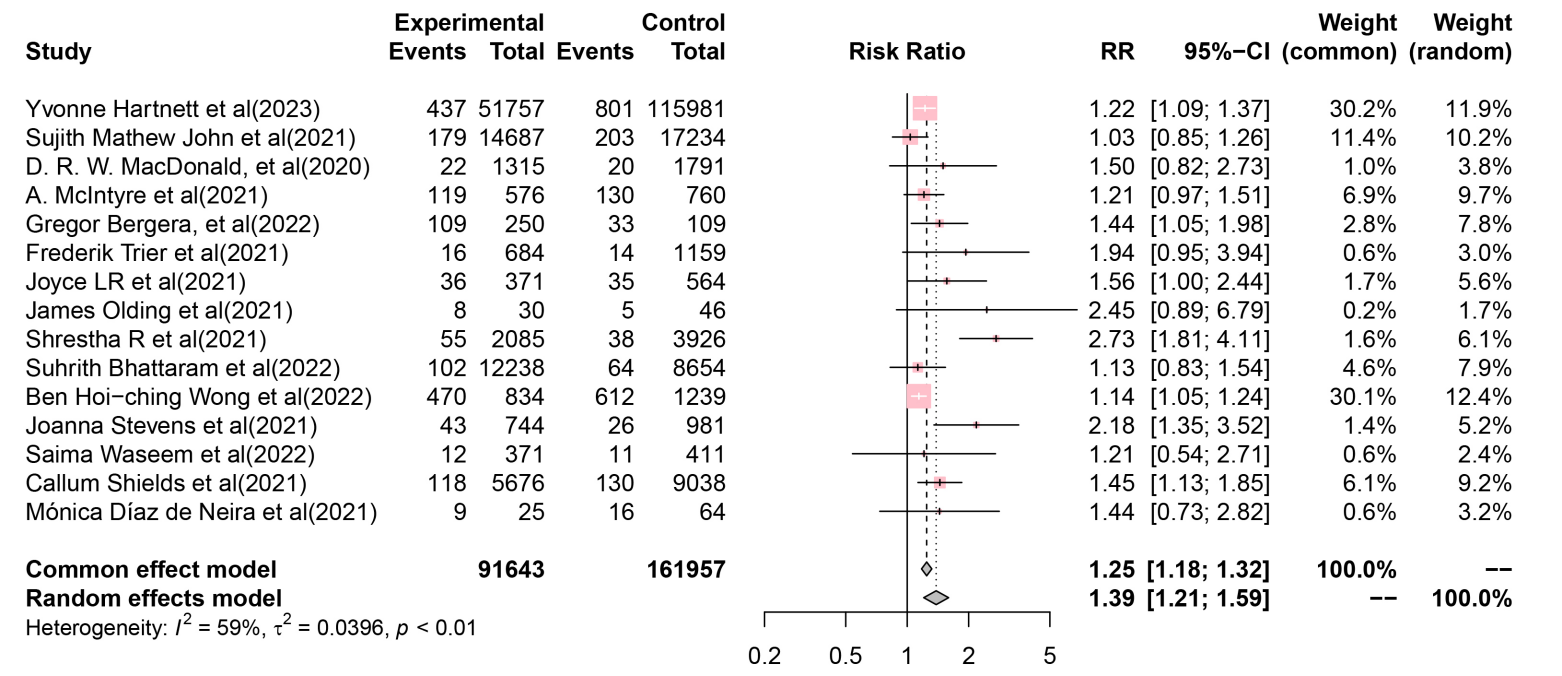
**Forest plot of Risk Ratio (RR) of self-harm incidence before and during the COVID-19 lockdown period**. The squares and diamonds represent individual studies and pooled RR values, respectively. The lines represent 95% confidence intervals for each main study.

The meta-analysis results provided strong evidence indicating an increased incidence of emergency department visits for self-harm during the COVID-19 lockdown period. Power calculations revealed that the sample size should be more than 10,112.22 (sig. level = 0.05, power = 0.9, alternative = two-sided) (**Supplementary Table 3**). The pooled RR of self-harm incidence before and during the COVID-19 lockdown period was 1.386 (95% CI, 1.205–1.595) (Fig. 2), which showed considerable heterogeneity (I^2^ = 58.9%, *p *= 0.002). Moreover, a considerable amount of between-study variance in the effect size remained (Q = 34.03, *p* < 0.001; I^2^ = 58.9%). Baujat-Galbraith plots were drawn to show the different contributions to heterogeneity (**Supplementary Fig. 6**). We performed several tests for publication bias, including funnel plots and Egger’s tests (*p* = 0.007). Considering the need for statistical significance, the trim-and-fill method using a random-effects model was applied to adjust for funnel plot asymmetry, and the pooled RR was 1.207 (95% CI, 1.015–1.435, *p *= 0.033). Influential analysis by conducting a leave-one-out analysis showed stable results for RR values and confidence intervals (**Supplementary Fig. 7**). Meanwhile, the pooled RR of mood disorder incidence before and during the COVID-19 lockdown was 1.571 (95% CI, 0.822–3.003) (**Supplementary Fig. 8**).

### 3.3 Subgroup Analysis of Self-Harm Incidence by Emergency Department Type, Age, and Other Factors

#### 3.3.1 Subgroup Analysis by Emergency Department Type

**Fig. 3.**
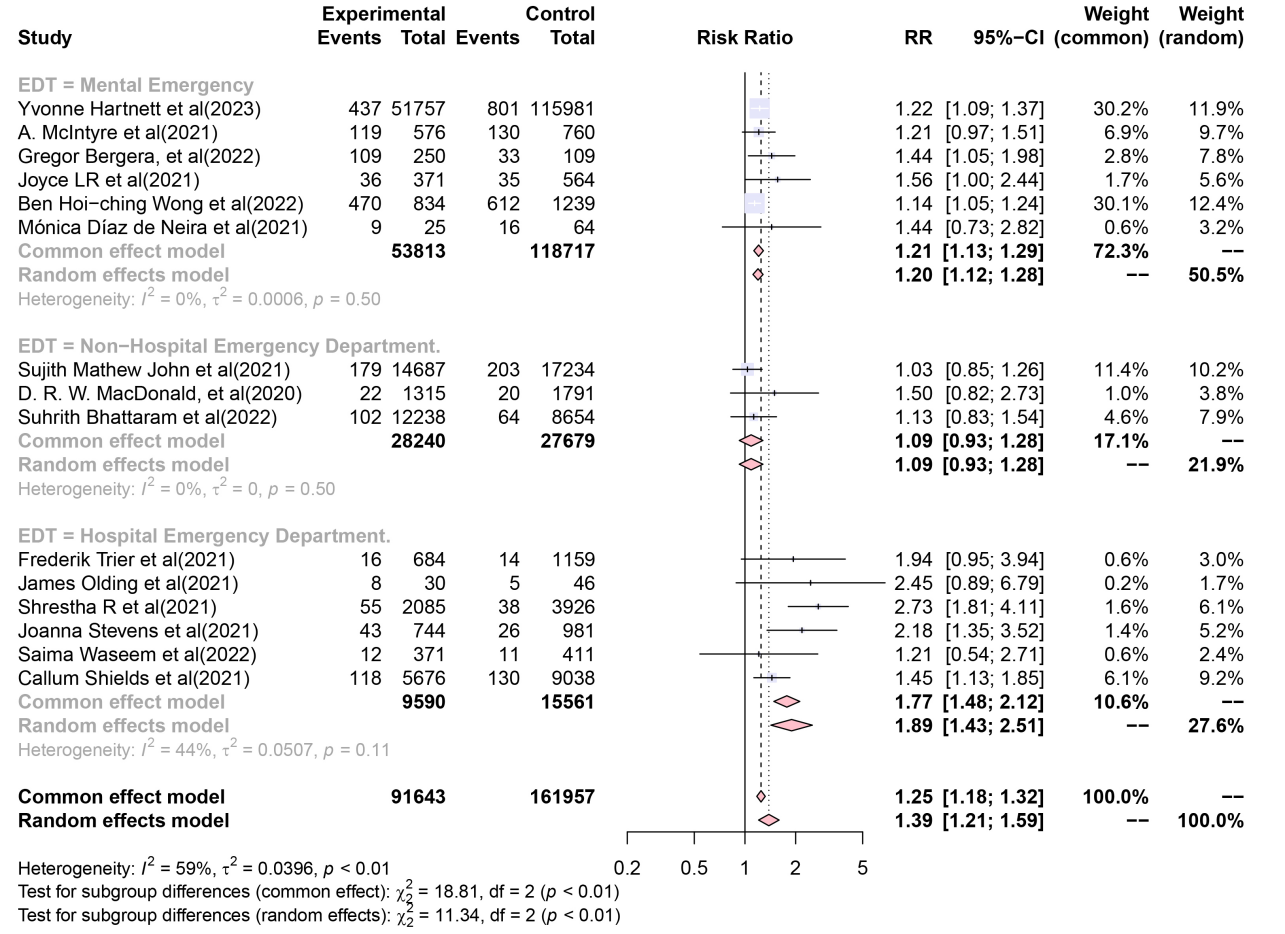
**Forest plot of subgroup analysis of “Emergency department type (EDT)”**. The squares and diamonds represent individual studies and pooled effect sizes, respectively. The lines represent 95% confidence intervals for each main study.

A random-effects model applied to a subgroup analysis of “Emergency department type (EDT)” produced pooled RR self-harm incidence values of 1.195 (95% CI, 1.116–1.281), 1.088 (95% CI, 0.925–1.279), and 1.892 (95% CI, 1.425–2.512) for Mental Emergency data, Hospital non-Emergency data, and Hospital Emergency Department, respectively (Fig. 3). The heterogeneity of RR values between subgroups of “EDT” (in the random-effects model) was significant (Q = 11.34, df = 2, *p *= 0.004).

#### 3.3.2 Subgroup Analysis by Age

**Fig. 4.**
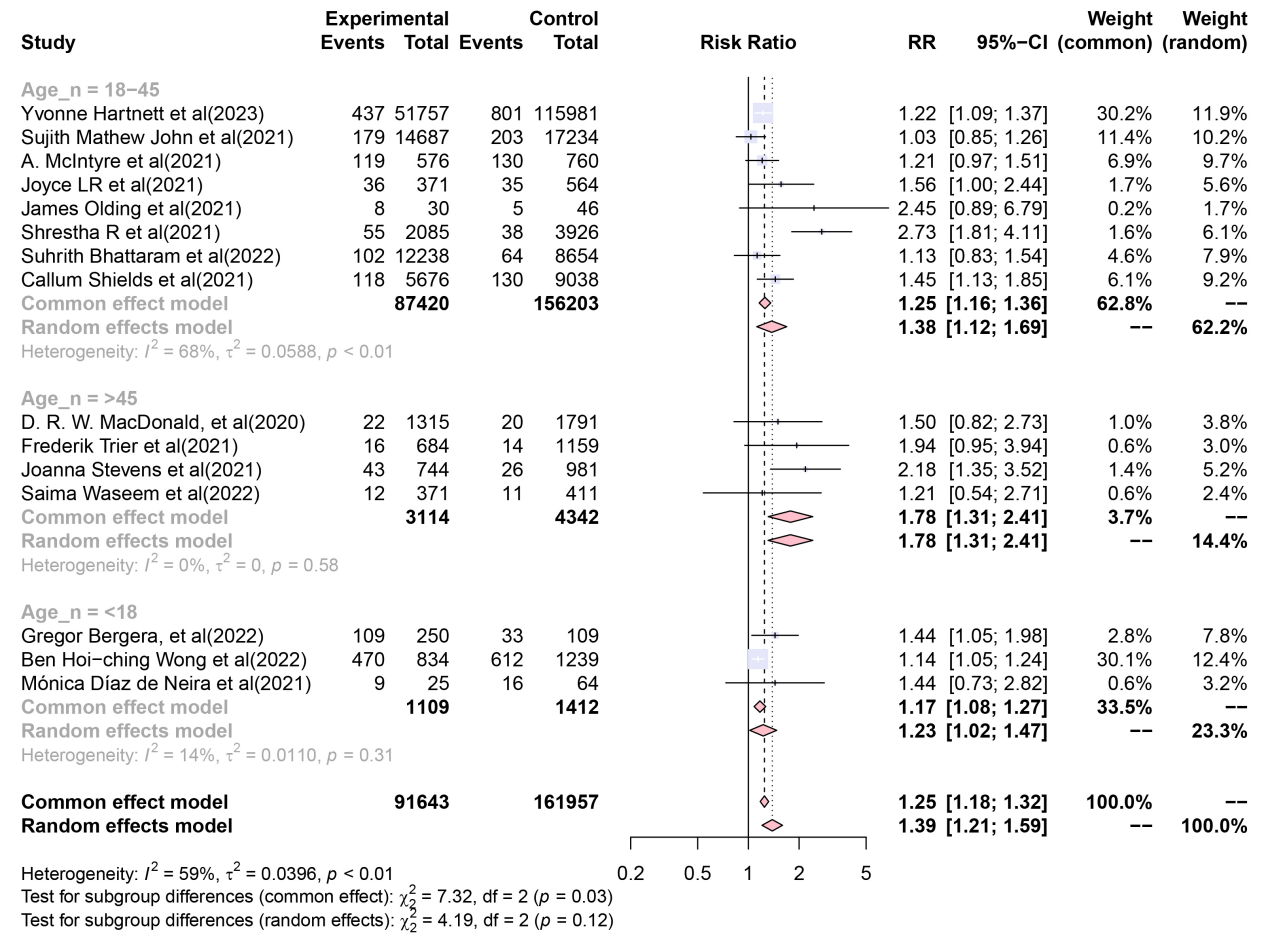
**Forest plot of subgroup analysis of the “Mean age of the sample”**. The squares and diamonds represent individual studies and pooled effect sizes, respectively. The lines represent 95% confidence intervals for each main study.

Additionally, applying a common-effects model, the subgroup analysis of the “Mean age of the sample” showed pooled RR self-harm incidence values of 1.254 (95% CI, 1.158–1.358), 1.778 (95% CI, 1.311–2.410), and 1.171 (95% CI, 1.081–1.268) for the “18–45”, “>45”, and “<18” age groups, respectively (Fig. 4). RR value heterogeneity between the subgroups of the “Mean age of the sample” (in the common-effects model) was significant (Q = 7.32, df = 2, *p *= 0.026). However, applying a random-effects model to this subgroup analysis resulted in a non-significant subgroup difference (Q = 4.19, df = 2, *p *= 0.123). These results imply that individuals from different age groups are dissimilarly affected by COVID-19 in terms of self-harm incidence.

#### 3.3.3 Subgroup Analysis by Other Factors

We also conducted a subgroup analysis on “Social economic status”, “Area”, “Statistic”, and “Definition”, using both the random-effects and the common-effects models. These differences between their respective groups were not statistically significant (Table 2).

**Table 2.** **RR value of self-harm incidence before and during COVID-19 among all samples according to different categories**.

Category	Subgroup	NO. of studies	RR [95% CI]	N	I^2^	*p*	
Total		15	1.39 [1.21–1.95]	253,600	59%		0.002
EDT	Mental Emergency	6	1.20 [1.12–1.28]	172,530	0%	0.50	<0.01
	Non-hospital Emergency Department	3	1.09 [0.93–1.28]	55,919	0%	0.50	
	Hospital Emergency Department	6	1.89 [1.43–2.51]	25,151	44%	0.11	
Age	<18	3	1.23 [1.02–1.47]	2521	14%	0.31	<0.05
	18–45	8	1.38 [1.12–1.69]	243,623	68%	<0.01	
	≥45	4	1.78 [1.31–2.41]	7456	0%	0.58	
SES	High-income	12	1.33 [1.19–1.48]	194,776	33%	0.13	>0.05
	Low-income	3	1.44 [0.80–2.60]	58,824	89%	<0.01	
Area	Europe	11	1.31 [1.17–1.47]	193,841	34%	0.12	>0.05
	Asia	3	1.44 [0.80–2.60]	58,824	89%	<0.01	
	Others	1	1.39 [1.21–1.59]	253,000			
Statistic	Prism	2	1.56 [0.89–2.72]	169,463	81%	0.02	>0.05
	SPSS	9	1.38 [1.12–1.69]	65,431	60%	0.01	
	Stats	2	1.31 [0.83–2.08]	3916	52%	0.15	
	Unknown	2	1.49 [1.17–1.89]	14,790	0%	0.32	
Definition	ICD-10	3	1.25 [1.12–1.39]	168,186	0%	0.59	>0.05
	Non-ICD	12	1.43 [1.19–1.72]	85,414	66%	<0.01	

Note: EDT, Emergency department type; CI, Confidence interval.

### 3.4 Meta-Regression Analysis by “Female Ratio”, “Sample Size”, and “Self-Harm Presentations”

The impacts of the female ratio, sample size, and self-harm presentations before and during the COVID-19 lockdown period were assessed by meta-regression analysis using a mixed-effects model. As predictors, “Female ratio” and “Sample size” did not significantly affect the pooled RR value of self-harm incidence. However, “self-harm presentations” before and during the pandemic were found to be significant moderators that contributed considerably to heterogeneity. The amount of heterogeneity they accounted for was 28.95% and 15.20%, respectively (Table 3).

**Table 3.** **Results for meta-regression analysis of RR value of self-harm incidence among all samples**.

Item	tau^2^	tau	I^2^	H^2^	R^2^	Test for residual heterogeneity	Test of moderators (coefficient 2)
Female ratio	0 (SE = 0.042)	0	0.00%	1.00	0.00%	QE (df = 5) = 1.676, *p* = 0.892	QM (df = 1) = 0.0826, *p* = 0.774
Sample size	0.0397 (SE = 0.042)	0.199	62.05%	2.64	0.00%	QE (df = 12) = 31.624, *p* = 0.002	QM (df = 2) = 3.5609, *p* = 0.169
Self-harm presentations (before)	0.0336 (SE = 0.026)	0.183	66.42%	2.98	15.20%	QE (df = 13) = 27.8094, *p* = 0.010	QM (df = 1) = 2.8702, *p* = 0.090
Self-harm presentations (during)	0.0281 (SE = 0.023)	0.168	61.28%	2.58	28.95%	QE (df = 13) = 24.721, *p* = 0.025	QM (df = 1) = 4.1110, *p* = 0.043

Note: SE, Standard error; QE, Q-statistic for heterogeneity.

## 4. Discussion

The results of this meta-analysis suggest some adverse effects of the COVID-19 pandemic and the related lockdown on the incidence of self-harm. The RR value for the entire analysis was 1.386 (95% CI, 1.205–1.595). After the precision adjustment, RR was 1.207 (95% CI, 1.015–1.435); therefore, we could presume that the population was more prone to self-harm during the lockdown period than before. The test for the subgroup differences in RR values between “EDT” groups was significant, which indicated that the most striking RR value was derived from the hospital emergency department group, with an RR of 1.892. The test for the subgroup differences in “Mean age of the sample” showed slight significance using the common-effects model, and the group of “>45 years” of age pooled the highest RR value of 1.778. The meta-regression test found that “Self-harm presentations” before and during the pandemic were significant moderators that contributed to heterogeneity, accounting for 28.95% and 15.20% respectively.

During the COVID-19 lockdown, individuals may have experienced increased stress, isolation, and uncertainty, which may have contributed to a higher risk of self-harm [29]. The limitations and restrictions imposed during lockdowns can disrupt daily life, social support networks, and access to physical and mental health resources, leading some people to resort to self-injurious and suicidal behaviors as a coping mechanism to express distress [35]. The level of self-perceived psychological resilience among the public may have been adversely affected by the continuing pandemic. Indeed, those who were less resilient expressed greater difficulty coping with the emotional challenges of the pandemic [36]. In addition, the COVID-19 pandemic could indirectly affect the outcomes of several physical diseases, such as cardiovascular diseases, through changes in human behavior and healthcare resource allocation, potentially leading to treatment delays [37]. There are interactions between brain maturation and the social environment, and isolation may impact the onset of psychiatric disorders [38]. Acute isolation can lead to social cravings, with Neural responses to cravings being similar to those of hunger, even at the neurofunctional level [39]. It is crucial to ask whether similar incidents occurred during the influenza pandemic of 1918–1919. There is some evidence that suicide deaths increased in the US [40], as well as among the elderly in Hong Kong during the severe acute respiratory syndrome (SARS) epidemic of 2003 [41].

Clinical institution presentations for self-harm incidence can be broadly divided into three categories: mental, hospital, and non-hospital emergency departments. Previous studies have shown an increase in the incidence of suicide and self-harm in hospital emergency departments during the initial phase of the COVID-19 pandemic [10, 11, 12, 13]. Similarly, the incidence of self-harm in nonhospital emergency departments has also increased [14]. Interestingly, in one non-emergency hospital study, overall injuries decreased by 35% compared to the same period in 2019, while self-harm incidence was significantly higher than in previous years (11% in 2019 and 2% in 2018) [26]. However, the observed increase in the incidence of self-harm in the psychiatric emergency department was not statistically significant, which might indicate that the stringency of the lockdown measures mediated the reduction in psychiatric emergency presentations [15]. Researchers have provided evidence showing that the rise in local COVID-19 cases is associated with a decrease in psychiatric and mental health emergency presentations in emergency departments [42, 43]. Nevertheless, other studies have found that mental-health-related self-harm presentations increased during the COVID-19 pandemic [25, 44]. In summary, it is worth exploring whether the type of clinical setting is a potential factor underlying self-harming behavior. The test for subgroup differences in “Emergency department type” was significant. The RR value for the “Hospital Emergency Department” was 1.892, which represented the most urgent and serious cases, revealing almost twice the self-harm incidence before the COVID-19 lockdown period. Amid the COVID-19 pandemic and lockdown, hospital visits dropped due to fear of infectious diseases and other inconveniences [45, 46]. One study during the COVID-19 period in the UK showed that despite the easing of restrictions, the overall admission rates in England, Scotland, and Wales remained lower by 20.8%, 21.6%, and 22.0%, respectively, compared to the same period (August-September) in the pre-pandemic years [47]. In addition, it can be inferred that due to the decrease in consultation rates, the hazard of self-harm became more prominent within the emergency visit population. The “Mental Emergency data” and “Non-Hospital Emergency Department” groups showed RRs of 1.195 and 1.088, respectively, which demonstrated that the psychiatric consultation group and the general population were both affected by COVID-19 partially, but not prominently.

Moreover, self-harm incidence was correlated with age. According to one study, 15% and 17% of college students and adolescents, respectively, self-harmed at least once [48]. Even though most attention has been paid to children and teenagers, self-harming behaviors can occur at any age. The incidence of self-harm among older adults has distinct characteristics that should be explored to improve management and care. Although the risk of further self-harm and suicide was high in all age groups, the risk of suicide was highest in older adults [49]. The issue of self-harm risk within the middle-aged and elderly demographics during the pandemic, not only among youth and adolescents, also warrants significant attention. The test for the subgroup difference of “Mean age of the sample” showed slight significance using the common-effects model. As a result, the group of “>45” years of age pooled the highest RR value, of 1.778. Compared to younger people and teenagers, middle-aged people and the elderly were more likely to be affected by the COVID-19 pandemic and the related lockdown [50]. A study published in 2022 systematically reviewed the mental health effects of the COVID-19 pandemic on older adults in China and found that the pandemic presented a threat to the physical and psychological health of middle-aged and elderly people [51]. Older adults are particularly vulnerable to the social impacts of the pandemic, including social distancing, if not outright social exclusion through quarantine, which exacerbates pre-existing loneliness, especially for those in residential care [52]. This emphasizes that more attention should be paid to the mental conditions of middle-aged and elderly people during the pandemic. Meta-regression analysis showed that “self-harm presentations” were significant moderators contributing to heterogeneity, suggesting that our study sample size was of great importance.

Our sub-study on mood disorder morbidity added valuable insights. We focused on the three studies among the 15 included studies that disclosed mood disorder morbidity. The pooled RR value of mood disorder incidence before and during the COVID-19 lockdown was 1.571 (95% CI, 0.822–3.003), which suggested that mood disorders might also increase during the pandemic to a certain extent; however, the result was not significant. Patients with preexisting psychiatric disorders reported worsening psychiatric symptoms during the COVID-19 pandemic. A variety of factors were associated with a higher risk of psychiatric symptoms and/or low psychological well-being, including female sex, poor self-related health, and relatives with COVID-19 [53]. In addition, high levels of posttraumatic stress were observed in participants who recovered from COVID-19, especially those who were symptomatic. Mild depression and anxiety were also reported [54].

This study has several limitations. First, the studies included in this meta-analysis were based on medical records that captured the relevant codes for presentations to medical facilities. As medical records vary, reporting is rarely standardized across sites, leading to measurement errors. Greater standardization of data reporting in this area is urgently needed so that common diagnostic tools, such as the ICD-11, can be used to specify outcomes across settings. Second, this meta-analysis did not determine the prevalence of suicidal ideation and behavior in the population. Further, it only excluded studies that did not distinguish between self-harm, suicidal ideation, and behavior. Third, this study focused on the “lockdown period” and included studies up to April 2022. With the change in policy, circumstances, and in-depth study of COVID-19, our results only represent a certain period. Although our results may bring some limited reflections, they do hold some promise for future studies. Quarantine to control the spread of COVID-19 remains complex. Not only do lockdowns have far-reaching health, social, political, and economic implications with both benefits and harms, but the benefits and harms of quarantine are unevenly distributed across a country’s population. Furthermore, the full effects of the quarantine have remained unknown for many years.

## 5. Conclusions

In conclusion, this study provides evidence of an increased self-harm incidence during the COVID-19 lockdown period. In addition, emergency department type and age have emerged as significant influential factors for self-harm incidence, particularly in patients from the Hospital Emergency Department and elders (age >45 years) who have a higher risk of engaging in self-harm. The results of this study have potential implications for future policy development and strategies for managing such situations.

## Availability of Data and Materials

The data are extracted from published studies and are available in the paper, and the datasets are not subject to restrictions.

## Supplementary Material

Supplementary material associated with this article can be found, in the online version, at https://doi.org/10.31083/AP39868.
