# Supplementary material for: The Effect of the COVID-19 Pandemic Lockdown on Self-Harm: A Meta-Analysis
Source: Alpha Psychiatry. 2025 Apr 22;26(2):39868. doi: 10.31083/AP39868 (PMC12059762; doi:10.31083/AP39868)
Supplement: Supplementary file 1 [file 2757-8038-26-2-39868-s1.zip › v4.2-Supplementary materia-final.docx]

**Supplementary materials**

**Search formulas for each database**：

APA PsycINFO: DE "COVID-19" OR DE "Coronavirus Disease 2019" AND DE "NSSI" OR DE "Non-suicidal self-injury" OR DE "Self-harm" OR DE "Self-injurious behavior" OR DE "Deliberate self-harm"

Embase: 'COVID-19'/exp OR 'Coronavirus Disease 2019'/exp AND 'NSSI'/exp OR 'Non-suicidal self-injury'/exp OR 'Self-harm'/exp OR 'Self-injurious behavior'/exp OR 'Deliberate self-harm'/exp

PubMed: ("COVID-19"[Mesh] OR "Coronavirus Disease 2019"[Mesh]) AND ("NSSI"[Mesh] OR "Non-suicidal self-injury"[Mesh] OR "Self-harm"[Mesh] OR "Self-injurious behavior"[Mesh] OR "Deliberate self-harm"[Mesh])

Web of Science: ("COVID-19" OR "Coronavirus Disease 2019") AND ("NSSI" OR "Non-suicidal self-injury" OR "Self-harm" OR "Self-injury" OR "Self-injurious behavior" OR "Deliberate self-harm" OR "DSH" OR "Self-cut")

CNKI, Wan Fang, and VIP (Chinese Databases): COVID-19 OR Coronavirus Disease 2019 AND NSSI OR Self-harm OR Self-injury OR Deliberate self-harm OR DSH OR Self-cut”

Supplementary Table 1. Quality assessment criteria.

| Criterion | Question |
| --- | --- |
| 1 | Was the research question or objective clearly stated? |
| 2 | Was the study population clearly specified and defined? |
| 3 | Were all the subjects selected or recruited from the same or similar populations (including the same time-period)? Were inclusion and exclusion criteria for being in the study prespecified and applied uniformly to all participants? |
| 4 | Was a sample-size justification, power description or variance and effect estimates provided? |
| 5 | Was the timeframe sufficient so that one could reasonably expect to see an association between exposure and outcome if it existed? |
| 6 | For outcomes that can vary in amount, type, or level, did they examine different levels of the exposure as related to the outcome (eg, categories of exposure, or exposure measured by a continuous variable |
| 7 | Were the exposure measures (independent variables) clearly defined, valid, reliable, and implemented consistently across all study participants? |
| 8 | Was the exposure assessed more than once over time? (eg, were there multiple post-COVID timepoints?) |
| 9 | Were the outcome measures (dependent variables) clearly defined, valid, reliable, and implemented consistently across all study participants? |
| 10 | Were key potential confounding variables measured and adjusted statistically for their impact on the relationship between exposure(s) and outcome(s)? |
| For each criterion, studies were given a score of 0 (criterion unmet) or 1 (criteria met). | |

Supplementary Table 2: Quality assessment scores of included studies.

| Source | Criterion | | | | | | | | | | Total |
| --- | --- | --- | --- | --- | --- | --- | --- | --- | --- | --- | --- |
|  | 1 | 2 | 3 | 4 | 5 | 6 | 7 | 8 | 9 | 10 | (0-10) |
| Yvonne Hartnett et al(2022) | 1 | 1 | 0 | 0 | 1 | 1 | 1 | 1 | 1 | 1 | 7 |
| Sujith Mathew John et al(2021) | 1 | 1 | 1 | 0 | 1 | 1 | 1 | 1 | 1 | 1 | 9 |
| D. R. W. MacDonald, et al(2020) | 1 | 1 | 1 | 0 | 1 | 1 | 1 | 1 | 1 | 1 | 9 |
| A. McIntyre et al(2020) | 1 | 1 | 1 | 0 | 1 | 1 | 1 | 1 | 1 | 1 | 9 |
| Gregor Bergera, et al(2022) | 1 | 1 | 1 | 1 | 1 | 1 | 1 | 1 | 1 | 1 | 10 |
| Frederik Trier et al(2021) | 1 | 1 | 1 | 0 | 1 | 1 | 1 | 1 | 1 | 1 | 9 |
| Joyce LR et al(2021) | 1 | 1 | 1 | 0 | 0 | 0 | 1 | 0 | 1 | 1 | 6 |
| James Olding et al(2020) | 1 | 1 | 1 | 0 | 0 | 0 | 1 | 1 | 1 | 1 | 7 |
| Shrestha R et al(2021) | 1 | 1 | 1 | 0 | 1 | 1 | 1 | 1 | 1 | 1 | 9 |
| Suhrith Bhattaram et al(2021) | 1 | 1 | 1 | 0 | 1 | 1 | 1 | 0 | 1 | 1 | 8 |
| Ben Hoi-ching Wong et al(2022) | 1 | 1 | 1 | 1 | 1 | 1 | 1 | 0 | 1 | 1 | 9 |
| Joanna Stevens et al(2021) | 1 | 1 | 1 | 0 | 1 | 0 | 1 | 0 | 1 | 1 | 7 |
| Saima Waseem et al(2021) | 1 | 1 | 1 | 0 | 1 | 1 | 1 | 1 | 1 | 1 | 9 |
| Callum Shields et al(2021) | 1 | 1 | 1 | 0 | 1 | 1 | 1 | 1 | 1 | 1 | 9 |
| Mónica Díaz de Neira et al(2021) | 1 | 1 | 1 | 0 | 0 | 1 | 1 | 0 | 1 | 0 | 6 |

Supplementary Table 3: Sample size estimation with the two-sample comparison of proportions power calculation.

| Items | n | P1(sum) | P2(sum) | sig.level | power | alternative |
| --- | --- | --- | --- | --- | --- | --- |
| Results | 10112.22 | 0.01893216 | 0.01320103 | 0.05 | 0.9 | two.sided |





s.Figure 1: The funnel plot of the RR value provided a visual representation of whether there were any remaining publications in this meta-analysis by testing the symmetry of the plot. Study estimates falling within the funnel shape were statistically slightly significant.





s.Figure 2: Trim-and-fill method was applied to adjust for funnel plot asymmetry and evaluated the influence of bias, after finishing the adjustment, the slight publication bias could be acceptable.



s.Figure 3: Egger’s test for publication bias assessment. Each data point represented an individual study included in the analysis, with the x-axis indicating the precision of the effect size estimate (represented by the inverse of the standard error) and the y-axis representing the standardized effect size estimate, which showed non-publication bias.





s.Figure 4: Begg’s test plot displayed the results of Begg's test. The symmetry of the funnel plot provided insights into a more comprehensive assessment of publication bias, with a symmetrical distribution potentially indicating non-bias.





s.Figure 5: Linear regression test displayed the meta-analysis results for linear regression models conducted across 15 studies. The line of best fit represents the overall trend across studies. The scatter of points around the line reflected heterogeneity among the studies. This plot provided evidence for a positive relationship between the COVID-19 pandemic and incidence of self-harm across the included studies."


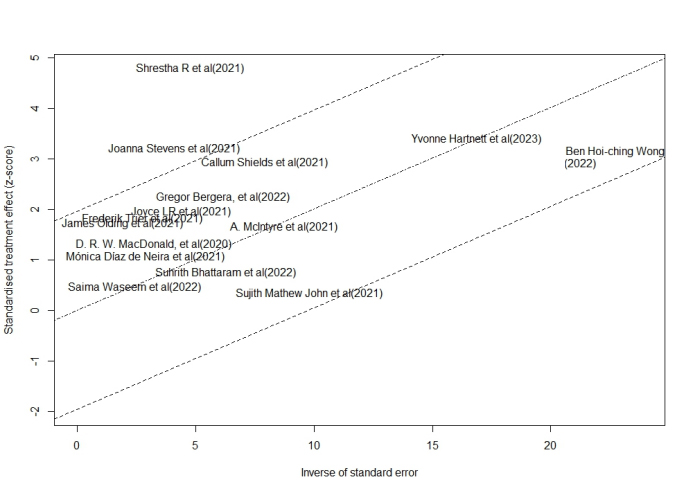


s.Figure 6: Galbraith plot displayed the results of a meta-analysis assessing the heterogeneity among 15 studies included in this analysis. The spread of the squares outside the Galbraith lines indicated the presence of heterogeneity among the studies. Studies with squares far from the center may be influential outliers contributing to the observed heterogeneity.


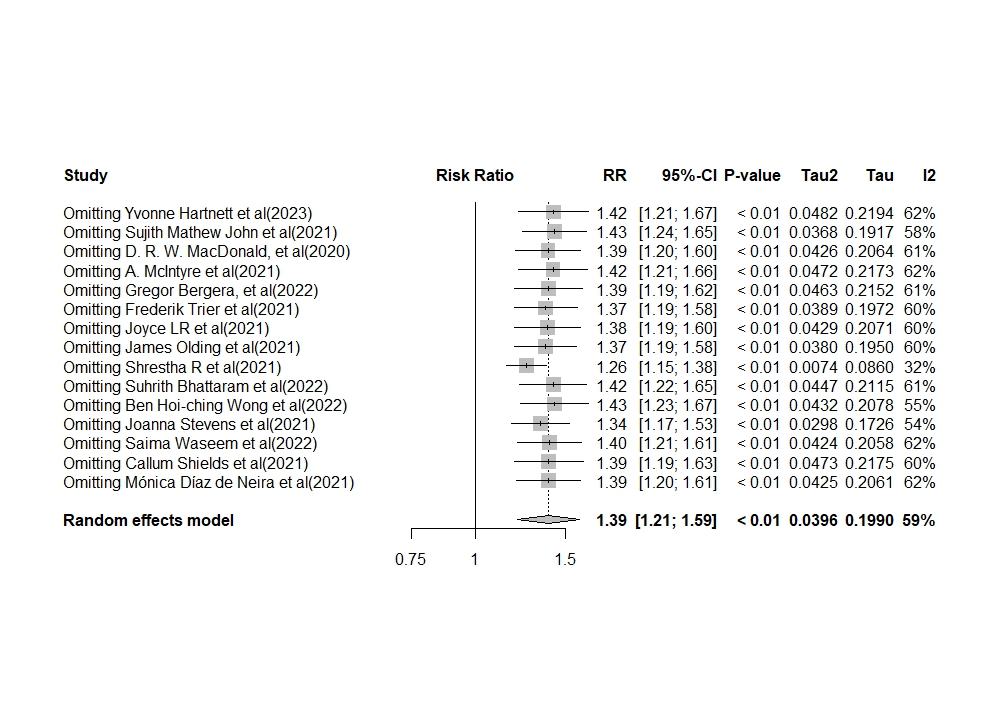


s.Figure 7: Leave-one-out analysis illustrated the sensitivity analysis conducted in the meta-analysis. Each point on the plot represents the meta-analysis result after systematically excluding one study at a time from the overall analysis. The Risk Ratio values and their associated 95% confidence interval are plotted for each iteration, which showed stable results.


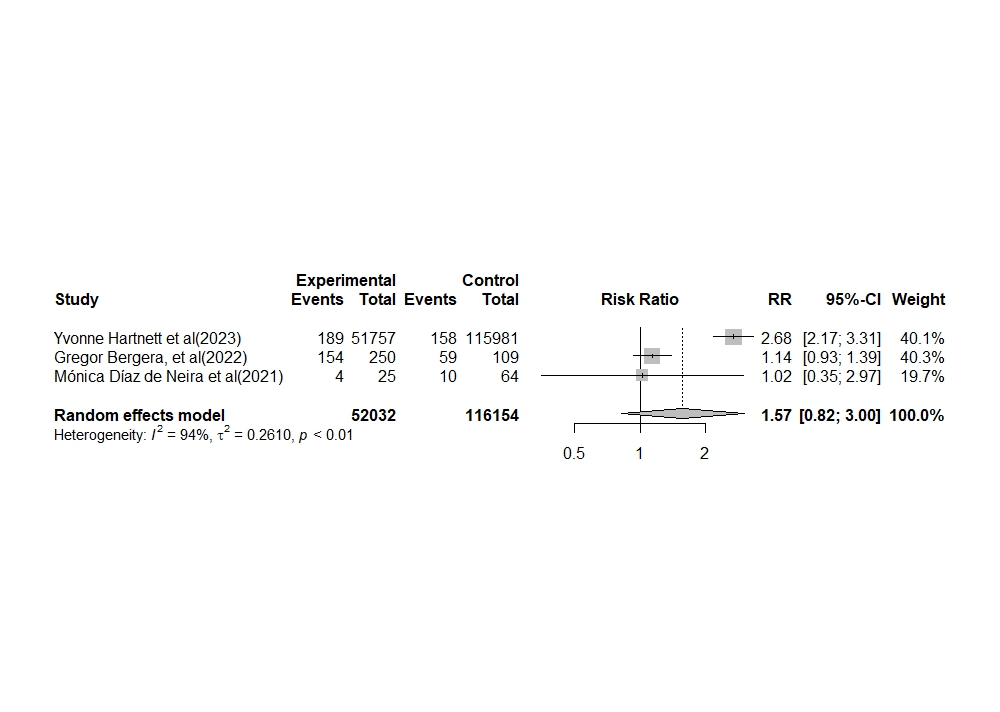


s.Figure 8: Mood disorder forest focused on the 3 studies that disclosed the mood disorder morbidity in the 15 included studies, the pooled RR value of mood disorder incidence before and during COVID-19 lockdown was 1.571(95% CI, 0.822-3.003), which suggested that mood disorders have also increased during the pandemic to a certain extent.
